# Supplementary material for: Henipavirus Matrix Protein Employs a Non-Classical Nuclear Localization Signal Binding Mechanism
Source: Viruses. 2023 May 31;15(6):1302. doi: 10.3390/v15061302 (PMC10303457; doi:10.3390/v15061302)
Supplement: Supplementary file 1 [file viruses-15-01302-s001.zip › viruses-2408416-supplementary.pdf]

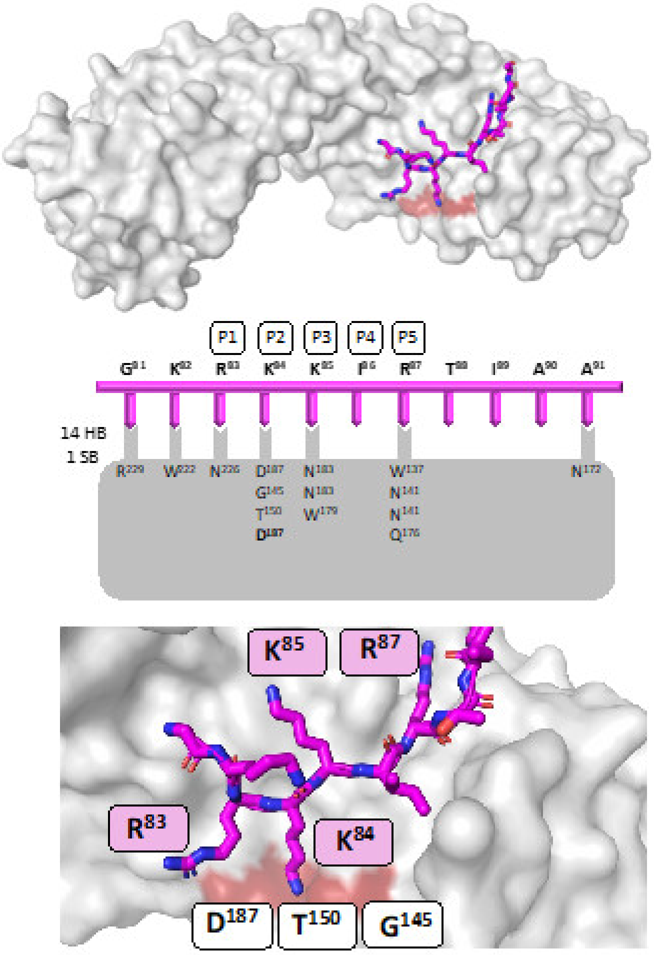

Figure S1. Structural basis for the interaction between Matrix putative NLS sequences to IMPα3. **A** HeV M NLS1 peptide sequence displayed in grey box with resolved amino acids highlighted in magenta. Matrix NLS1 (magenta sticks) binds to IMPα (grey surface) at the major site. **B** Schematic of the interface between NLS1 (magenta) and IMPα (grey). HeV M K^{84} binds to P2 site on IMPα (Gly145, Thr150, and Asp187) (highlighted red) with H-bonds and salt bridge interactions depicted. **C** The resolved amino acids of HeV M NLS1 (magenta sticks) are shown in the major binding site of IMPα.
